# Assessing Unmet Social Needs in Multiple Sclerosis Care in Australia: A Qualitative Assessment of Feasibility, Barriers and Enablers

**DOI:** 10.1111/hex.70691

**Published:** 2026-05-18

**Authors:** Megan R. Hawkins, Yvonne C. Learmonth, Isabelle Weld‐Blundell, Darshini Ayton, Jodi Haartsen, Anne Kavanagh, Tomas Kalincik, Marlena Klaic, Claudia H. Marck

**Affiliations:** ^1^ Disability and Health Unit, Centre for Health Policy, Melbourne School of Population and Global Health The University of Melbourne Melbourne Victoria Australia; ^2^ School of Health Sciences The University of New South Wales Sydney New South Wales Australia; ^3^ School of Allied Health Murdoch University Perth Western Australia Australia; ^4^ Neuroscience Research Australia Randwick New South Wales Australia; ^5^ Perron Institute for Neurological and Translational Science Perth Western Australia Australia; ^6^ School of Public Health and Preventive Medicine Monash University Melbourne Victoria Australia; ^7^ Alfred Health Monash University Melbourne Victoria Australia; ^8^ Neuroimmunology Centre, The Royal Melbourne Hospital Melbourne Victoria Australia; ^9^ CORe, Department of Medicine The University of Melbourne Melbourne Victoria Australia; ^10^ Melbourne School of Health Sciences The University of Melbourne Melbourne Victoria Australia

**Keywords:** chronic disease, health inequalities, patient‐centred care, social determinants of health, social prescribing, social support

## Abstract

**Background:**

Unmet social needs (including housing, transport and social inclusion) contribute substantially to health outcomes, especially for people with long‐term health conditions such as multiple sclerosis (MS). Whether assessment of unmet social needs occurs in MS clinical care is unclear. This study aims to (1) understand current practices, (2) identify barriers and enablers to social needs assessments in MS care, and (3) explore the feasibility of social needs screening tools.

**Methods:**

This qualitative descriptive study comprised focus groups and interviews with clinicians, carers and people with MS in Australia. We inductively and deductively coded transcripts and used applied thematic analysis to identify themes, using COM‐B (Capability, Opportunity, Motivation, Behaviour) and Feasibility frameworks.

**Results:**

We collected data from 19 participants (11 clinicians and 8 lived‐experience participants). Participants reported inconsistent and unstructured social needs assessment in MS care. Barriers aligned with capability (lack of referral pathway knowledge), opportunity (resources) and motivation (belief in abilities, evidence of value and scepticism). Enablers aligned with capability (clinician rapport and self‐advocacy), opportunity (care coordination and self‐paced assessment), and motivation (mutual desire for positive patient outcomes). A social needs screening tool was considered acceptable, conditional on adequate resourcing and/or design to ensure identified needs can be addressed.

**Conclusion:**

Findings from this qualitative descriptive study suggest that the current integration of social care in MS healthcare in Australia is inadequate. We identified opportunities to develop and test a screening tool and supporting referral resources to identify and address unmet social needs in MS patients. Better integrated social and healthcare represents a promising avenue for improving comprehensive MS management.

## Patient or Public Contribution

The author team comprised people living with MS and people working in MS care or MS research. All authors contributed to the design of the study, including the focus group materials, the interpretation of the data during the analysis, and the reporting of the findings through reviewing and editing the manuscript.

## 1 Introduction

Multiple sclerosis (MS) is a chronic neurological condition commonly diagnosed in the third and fourth decades of life [1]. MS affects more than 33,300 Australians and over 2.9 million people worldwide [1] and often necessitates multidisciplinary healthcare due to its wide range of physical, cognitive and mental symptoms. The integration of social and clinical care has been highlighted as an important part of the patient–neurologist relationship [2, 3] and is associated with health outcomes [4, 5, 6]. Inequalities in access to health and social services have been identified in North American and European settings among people with MS, with suboptimal access experienced by those from lower socio‐economic backgrounds, lower levels of education, non‐Caucasian ethnicities, and rural communities [7]. In Australia, MS state‐based charitable organisations and the National Disability Insurance Scheme (NDIS), which provides funding and support to eligible individuals with permanent and significant disabilities, play a key role in providing social services to people with MS. However, it is unclear to what extent people with MS are receiving integrated clinical and social care in Australia.

Social determinants of health describe the conditions in which people are born, live and work, and their access to power, money and resources [8]. Social determinants of health are estimated to account for 30%–55% of health outcomes [8]. The extensive influence of social determinants of health produces profound disparities among people with MS, manifesting in unequal access to early diagnosis and timely medical interventions, ultimately shaping divergent long‐term health trajectories [4, 5, 6]. Social needs are the acute, non‐medical needs that result from underlying social determinants of health [9], such as access to adequate nutritious food, affordable quality housing, and employment or income security. Addressing unmet social needs through social care may reduce health inequities and improve health [8, 10]. Social inequities are shaped by macro‐level determinants, including the political, economic and cultural environments that are formed by policies at the local, national and global levels [11, 12]. Macroeconomic policies, such as the national income support system and NDIS, play a role in supporting the social needs of Australians more broadly [13, 14]. However, not all people with disability and chronic conditions, including MS, benefit from these initiatives, and inequalities can remain even for those who do [15].

Integrating health and social care (e.g., through screening for unmet social needs in healthcare settings) has shown promise for improving health outcomes for patients in the United States and the United Kingdom [16, 17, 18]. Interventions in clinic waiting rooms linking patients to social services showed increased social service use and reductions in unmet social needs [19, 20]. Social needs interventions or social prescribing in healthcare settings may improve health and well‐being through: (1) reducing unmet social needs; (2) reducing chronic stress and anxiety; (3) improving quality of care and care effectiveness; and (4) reducing care provider burnout [21]. Our recent scoping review (under review) identified five social needs screening tools developed for adult clinical populations in Australia. For example, the Flinders University Screening Tool (FUST) was piloted in people with chronic lung or heart conditions or diabetes living in a disadvantaged urban Australian population and demonstrated benefits for both clinicians and patients.

Social needs assessments in healthcare settings may be especially suitable for people who are more likely to have unmet social needs, such as people with disabilities or chronic health conditions, such as MS [22]. Italian research showed that 68% of MS patients had unmet social needs linked to reduced quality of life [23], with individualised assessment needed since traditional risk factors (age, education, disease duration and disability) do not fully predict these needs [24]. An Australian study from 2006, which interviewed people living with MS and their carers attending a tertiary hospital, identified that 39% of participants reported difficulty accessing rehabilitation interventions for MS, 15.8% reported difficulties with work and 5% reported difficulties with transport [25]. While they reported on some unmet social needs, it was not the primary purpose of this study. Research focussing on comprehensive social needs assessment or social care interventions in the Australian clinical MS care setting is mostly lacking.

This study aims to: (1) understand current practice in the Australian MS clinical care setting related to assessing and addressing social needs; (2) identify the barriers and enablers to social needs assessment in this setting; and (3) explore the feasibility of social needs screening tools, from the perspectives of both clinicians and consumers.

## 2 Materials and Methods

We used established frameworks to address the study aims. The COM‐B behavioural science framework was proposed in the setting of healthcare implementation research and describes three essential conditions for a behaviour: Capability, Opportunity and Motivation [26]. Identifying deficits in these conditions can assist in designing interventions to change behaviour. The feasibility framework by Bowen et al. [27] is a public health implementation framework, proposing eight areas of focus for feasibility studies: acceptability, demand, implementation, practicality, adaptation, integration, expansion and limited‐efficacy testing. We used the first six domains of the Bowen feasibility framework to explore clinician and people with lived experience of MS (consumer) attitudes towards the feasibility of existing social needs screening tools to help inform future directions [27]. This descriptive qualitative study used a pragmatic and exploratory approach and was reported in accordance with the Consolidated Criteria for Reporting Qualitative Research (COREQ) [28].

### 2.1 Ethics

This study was approved by the University of Melbourne Human Ethics Committee (2025‐31423‐63802‐3). We obtained informed consent from participants at two time points: initially via an electronic form on Qualtrics (Provo, Utah, the United States), followed by verbal confirmation at the beginning of each focus group or interview.

### 2.2 Research Team and Reflexivity

The research team comprised MS researchers with expertise in qualitative methods (CHM [female, PhD], YCL [female, PhD], IWB [female, MD]), social science researchers living with MS (AMK [female, MD, PhD], DA [female, PhD]), and clinician‐researchers (TK [male neurologist], JH [female nurse], MH [female physiotherapist], MK [female occupational therapist, PhD], YCL [female physiotherapist]). Many of the team have a longstanding interest in reducing social and health inequalities, in particular for people with disabilities or long‐term health conditions such as MS. There was no personal relationship between any of the interviewers and participants, and participants did not have any knowledge about the interviewers' views prior to study commencement.

### 2.3 Participants and Recruitment

We used purposive sampling with snowballing to recruit MS clinicians and consumers. We intended to recruit approximately eight MS clinicians from a variety of specialties (nursing, neurologists and various allied health) and eight lived‐experience participants (people with MS and/or carers for people living with MS), with the aim of conducting two focus groups with four participants for each group. We recruited clinicians via social media advertising (e.g., LinkedIn), emails to professional networks (e.g., neurology colleagues of collaborators), and clinicians who had previously expressed interest in participating in MS research. We recruited consumers via social media advertising, a lived‐experience network (MS Australia Lived Experience Expert Panel), and consumers who had previously expressed interest in participating in MS research.

Interested participants provided consent and demographic information via the online survey (Qualtrics). We conducted focus groups in the first instance to promote synergistic effects between participants to deepen understanding, idea generation and comprehensiveness of insights. Focus groups were scheduled based on mutual participant availability; if a participant withdrew from the study, another participant from the same subgroup (clinician or consumer) was offered a place in the focus group, where possible. Interviews were conducted as an alternative for a small number of clinicians who were not available to attend focus groups due to clinical commitments, to ensure we captured a diverse range of clinician specialities and gender. We reimbursed focus group, paired interview and individual interview participants with $120, $120 and $65 shopping vouchers, which reflected respective time commitments. Following the focus group/interview, we sent participants a list of services that provide support for social needs via email.

#### 2.3.1 Eligibility

Clinician participants were eligible to participate if they were currently or previously working regularly with people with MS as a neurologist, nurse or allied health professional. Consumer participants were eligible to participate if they self‐reported a diagnosis of MS or caring for a person with MS and had experience with one or more unmet social needs (e.g., housing, transport and income). As focus groups and interviews were conducted via videoconference and in English, participants were required to have sufficient spoken English‐language skills and the capability to use the Zoom online platform to participate. Only participants located in Australia were eligible.

### 2.4 Data Collection

We designed a focus group and interview discussion guide to explore the research aims. Topics in the discussion guide included current experiences of social needs assessment, barriers and enablers to social needs assessment, and feasibility of a social needs screening tool (informed by the framework by Bowen et al. [27]). Study investigators—including clinicians, researchers and people with MS—reviewed the focus group discussion guide to further refine the questions. C.H.M. and M.R.H. conducted all focus groups and interviews.

We collected data from participants through focus groups and semi‐structured interviews between May and July 2025. Focus groups and interviews were audio recorded, auto‐transcribed, manually checked for accuracy, and uploaded to NVivo Version 15 [29] for data management. No field notes were made during the interviews or focus groups, and no repeat interviews were conducted. To prompt further data collection, we sent participants a content summary of their interview or focus group (generated using a secure university‐specific AI tool after de‐identifying the transcript and then edited by the researcher who attended the focus group or interview) within 24 h and asked participants to provide any additional comments within 2 weeks if they wished, via email.

#### 2.4.1 Focus Groups

We conducted separate focus groups for clinicians and consumers via Zoom Workplace (Version: 6.5.9), with two to four participants and two facilitators per group. We intended to recruit four participants to each focus group and deliberately kept this number small due to the sensitive nature of the topic and to ensure adequate opportunity for each participant to voice their perspectives in the limited time. Prior to attending the focus group, we asked participants to review the plain‐language materials (available in written or video format) on social determinants of health. These materials included two Australian social needs screening tools [30, 31], deemed by our study investigators as the most comprehensive, relevant and feasible for the MS care setting, sourced from our scoping review [32]. Facilitators introduced themselves (i.e., senior MS researcher and research assistant) and the research aims at the beginning of each focus group.

#### 2.4.2 Interviews

We conducted semi‐structured interviews for a subgroup of clinicians who were unable to attend a focus group. We used the same interview guide for the semi‐structured interviews as the discussion guide for the focus groups.

### 2.5 Data Analysis

We conducted applied thematic analysis to identify themes relevant to the research questions [33]. Text was segmented and inductively coded to generate an initial codebook, with related codes grouped into categories. A subsequent round of deductive coding explored barriers and enablers, guided by the COM‐B domains of capability, opportunity and motivation [26]. Inductive and deductive codes were used to identify emergent themes; themes related to barriers and enablers of screening for social needs were ultimately mapped to the COM‐B framework.

Feasibility themes were deductively coded using Bowen et al.'s framework [27] of eight key areas of focus for feasibility studies in public health. M.R.H., C.H.M. and Y.C.L. led coding and theme development, with all study investigators reviewing through online meetings. The coders met regularly, and where there were discrepancies, these were discussed and resolved, and the codebook was updated accordingly. Themes and codes were presented on multiple occasions to the collaborator group, who provided feedback to ensure themes and code descriptions were relevant. We determined sufficient data collection for each subgroup (clinicians and consumers) when our analysis resulted in very few changes to the codebook, and this was achieved by our final transcripts, where the number of new codes considerably diminished. Quotes were abridged for conciseness and de‐identified; all quotes reported in this paper have come from focus groups, unless otherwise specified.

## 3 Results

**Figure 1:**
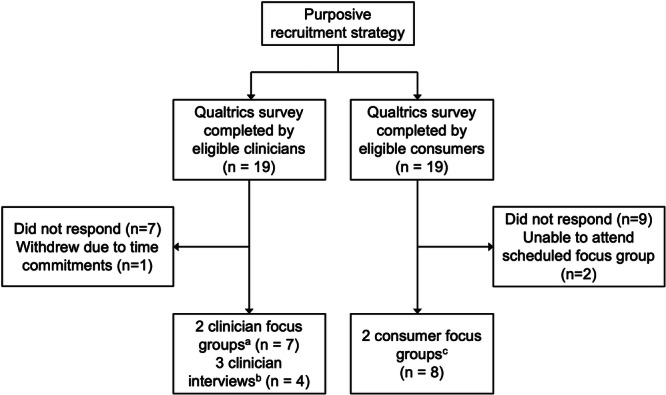
Participant flow from study recruitment to focus group/interview. ^a^Two clinician focus groups comprised: one allied health professional and two MS nurses (*n* = 3), two allied health professionals and two MS nurses (*n* = 4). ^b^Three clinician interviews comprised: neurologist (*n* = 1), neurologist (*n* = 1) and allied health professional (from subgroup not already captured in focus groups) (*n* = 2). ^c^ Two consumer focus groups comprised: three people living with MS and one carer for a person with MS (*n* = 4), and four people living with MS (*n* = 4).

We conducted three clinician focus groups, two consumer focus groups and two individual clinician interviews via Zoom to collect data from 11 clinicians and 8 consumers (Figure 1). Focus groups were 83–90 min in length, and interviews were 29–34 min in length. The size of the focus groups ranged between two and four participants. Three participants provided additional comments via email after their focus group.

Clinician participants included nurses, physiotherapists, neurologists, social workers and one dietitian (Table 1). The median age of participants was 35 years (IQR: 33–51, range: 25–56). Consumer participants included seven people with MS and one carer. The consumer participants' levels of disability were predominantly mild to moderate, with a median Patient‐Determined Disease Steps score of 2.5 (IQR: 2–4, range 0–6). In one focus group, one non‐participant was present to support a consumer in their role as disability support worker, and while they consented to participate, they did not contribute to the discussion and were not included as a participant in the results.

**Table 1:** Socio‐demographic characteristics of participants.

Characteristic	Clinician (*n* = 11)	Consumer (*n* = 8)
Age (median, IQR)	35 (31, 52)	49 (42, 54)
Gender		
Female	8 (73%)	6 (75%)
Male	3 (27%)	2 (25%)
Ethnicity		
North‐West European	2 (18%)	3 (38%)
Oceanian	5 (45%)	1 (13%)
Prefer not to say	1 (9%)	1 (13%)
Prefer to self‐describe	1 (9%)	2 (25%)
South and Central Asian	0 (0%)	1 (13%)
South‐East Asian	2 (18%)	0 (0%)
State		
New South Wales	3 (27%)	3 (38%)
Queensland	1 (9%)	1 (13%)
Tasmania	1 (9%)	1 (13%)
Victoria^a^	6 (55%)	3 (38%)
Rurality		
MM1 (major cities)	11 (100%)	5 (63%)
MM2 (regional centres)	0 (0%)	2 (25%)
MM4 (medium rural towns)	0 (0%)	1 (13%)
Consumer type		
Carer for person with MS	—	1 (13%)
Person living with MS	—	7 (88%)
Patient‐Determined Disease Steps		
0 (no disability)	—	1 (13%)
1 (mild disability)	—	1 (13%)
2 (moderate disability)	—	2 (25%)
3 (gait disability)	—	2 (25%)
6 (bilateral support, e.g., crutches)	—	2 (25%)
Healthcare profession		
Allied health	5 (45%)	—
MS nurse	4 (36%)	—
Neurologist	2 (18%)	—
Years of clinical practice (median, IQR)	10 (5, 19)	—
Number of patients with MS seen per week (mean, SD)	13 (7)	—
Work setting^b^		
Not‐for‐profit	4 (36%)	—
Not‐for‐profit and public	1 (9%)	—
Private	1 (9%)	—
Public	5 (45%)	—

*Note:* Normally distributed continuous variables are presented as mean (standard deviation), skewed continuous variables are presented as median (interquartile range), and categorical variables are presented as number (proportion of subgroup, i.e., proportion of clinicians or consumers). Note that some proportions of subgroups do not add to 100 due to rounding to zero decimal places. All consumers were unrelated to one another, i.e., the carer for a person with MS did not care for any of the people living with MS who participated in this study.

Abbreviations: IQR, interquartile range; MM1‐4, Modified Monash Model category [34]; MS, multiple sclerosis; SD, standard deviation.

^a^ Three Victorian clinicians also provided services to people living with MS in New South Wales, Tasmania and Australian Capital Territory via telehealth.

^b^ Information not collected as part of the Qualtrics survey (collected during focus group/interview).

First, we provide an overview of current practices with perspectives from clinicians and consumers, followed by the exploration of barriers and enablers to social needs screening in people with MS. The final section explores attitudes on the feasibility of a social needs screening tool.

### 3.1 Current Practices of Social Needs Screening

Key themes related to clinician and consumer experiences of current social needs screening and follow‐up practices are summarised in Table 2. Additional quotes are provided in Supplementary Table 1.

**Table 2:** Barriers and enablers to social needs assessment in MS care, mapped to the COM‐B^a^ capability domain.

		Theme (clinician)	Theme (consumer)
Capability	Barrier	Lack of knowledge of available resources	Cognitive capacity (fatigue, anxiety)
	Enabler	Patient clinician rapport (including longevity and continuity of care)	Self‐advocacy
Opportunity	Barrier	Lack of resources (time, space, staff)	Lack of access to MS nurses
		Coordination of care	Flexible, self‐paced completion
	Enabler	Coordination of care	Access to a multidisciplinary team
Motivation	Barrier	Lack of belief in the capability to provide solutions	Scepticism about clinician follow‐up
		Lack of evidence of value	Scepticism about the availability of resources (belief about consequences, pessimism)
			Privacy concerns
	Enabler	Benefits of data collection (belief)	Desire for disclosed social needs to be addressed
		Part of the health professional's role to refer and screen (identity)	
		Intention to provide holistic care and to action identified needs (intention)	
		Improved patient outcomes (belief)	
		Structure and consistency (belief)	
		Benefits of early screening (belief)	

^a^ Barriers and enablers mapped to the COM‐B framework [26].

#### 3.1.1 Clinician Experiences

Clinicians reported that no formal screening tools existed to identify social needs in people with MS, as well as inconsistencies in informal screening processes within and between clinics. Many clinicians reported referring people with MS to services addressing social needs, for example, state‐based MS organisations, Housing Connect, National Disability Insurance Scheme (NDIS), food delivery, and legal assistance. However, the use of these referral pathways was variable within and between health services and commonly depended on the individual clinician having some knowledge of their existence. Further, many indicated that social need referrals were only made after an escalating incident or a crisis, such as homelessness or domestic violence, emerged. Clinicians told us the existing practice for social needs assessment was insufficient in pre‐emptively identifying unmet patient needs:

Certainly it is our, any healthcare professional role … however are we doing it sufficiently? Probably not

clinician 4, nurse

#### 3.1.2 Consumer Experiences

Similar to clinicians, consumers reported that social needs were not routinely or comprehensively assessed. Consumer examples highlighted high variability in comprehensive care and care coordination, with one participant describing well‐coordinated support as inpatient, compared to an outpatient:

*‘If you end up in hospital with a flare … you've got a multidisciplinary team in the hospital, [as] opposed to a singular person … and they can engage with other areas in the hospital to get your needs seen better’.*

consumer 5

While other consumers also reported experiences of poor care coordination:

*‘I find everyone passes the buck to the next person. So my neurologist always says to me, “Your GP needs to take care of these issues.”’*

consumer 6

Poor access to services, poor‐quality care and ineligibility for support services, such as NDIS, were reported to impact consumers' experiences of social needs supports. Variability was commonly reported based on the state or how far from a metropolitan area an individual lived. Consumers commonly reported that management of social needs required high levels of self‐advocacy, health literacy and the ability to seek out resources independently, which created concern for some people with MS who might not have the resources or abilities to do this.

### 3.2 Barriers and Enablers to Social Needs Screening

We identified capabilities, opportunities and motivators (related to the COM‐B framework) that supported or hindered the behaviour of social needs screening, indicated in Table 2. Additional quotes are provided in Supplementary Tables 2, 3 and 4.

#### 3.2.1 Capability (Clinicians)

A capability barrier identified by clinicians was a lack of knowledge of the unmet social needs people with MS may experience, available resources and referral pathways to address these needs. Clinicians reported that a lack of confidence or knowledge of how to respond when a social need was identified was a barrier to assessing and/or effectively addressing social needs:

Asking the question is rather easy once you're trained … knowing what to do with that and where to lead people with that, at this point in time, I wouldn't feel confident.

clinician 1, allied health

In contrast, patient–clinician rapport, fostered by interpersonal skills, long‐term relationships in MS care and continuity‐focused models, was perceived as an important capability enabler.

#### 3.2.2 Capability (Consumers)

When we presented consumers with social needs screening tools, they identified common symptoms of MS, including fatigue and anxiety, as key capability barriers to engaging with these tools, especially in the context of a clinic appointment where consumers might have difficulty processing information. Consumers identified that self‐advocacy skills, a psychological capability enabler, allowed them to either speak up for themselves or to actively seek support for social needs through the ability to make informed decisions:

I'm fortunate enough to understand and know about support workers … but a lot of people don't know this stuff

consumer 3

#### 3.2.3 Opportunity (Clinicians)

A key opportunity barrier to screening for social needs in MS care was a lack of resources, including time, staff and physical space to appropriately complete screening and follow‐up and *handle it sensitively (clinician 8, neurologist, interview)*.

Concern regarding lack of opportunity for people with MS to complete a screening tool privately—either because of the need for assistance, lack of privacy at home or the nature of *very crowded waiting rooms (clinician 8, neurologist)—*was also expressed as a physical barrier by some clinicians:

Because of some of the sensitivity of the questions, for some people, they might not want to complete it in the home environment

clinician 5, allied health

The lack of social worker availability in the MS care setting was also raised by clinicians as a concern relating to the ability to address social needs, making it *harder to refer on (clinician 5, allied health)*.

Some clinicians identified a lack of coordination of patient care between clinicians or between hospitals and community services as a barrier to addressing social needs, stating that *the community‐hospital link is not very good (clinician 11, allied health, paired interview)*.

Conversely, other clinicians reported that good relationships between multidisciplinary teams and between hospitals and community services were an enabler to addressing social needs, but these links did not appear to be a consistent experience across clinicians or services.

We've got a multidisciplinary team meeting after clinic and this is where a lot of this stuff gets raised.

clinician 8, neurologist, interview

#### 3.2.4 Opportunity (Consumers)

The key opportunity barrier identified by consumers related to social needs screening in MS was a lack of access to MS nurses. Several consumers reported that an MS nurse was an appropriate person to complete social needs screening, while also stating *we need more MS nurses around (consumer 4)* and *I have never seen an MS nurse (consumer 3)*. The lack of rapport in some consumer–clinician relationships reported by consumers also served as a potential capability barrier perceived by consumers:

If I talk to professionals, certain professionals, it really feels like I'm talking … but I'm not really heard

consumer 3

Completion of a social needs screening tool *at home (consumer 3)* and *in my own time (consumer 8)* was identified as a potential opportunity enabler. Consumers reported that the opportunity to complete a self‐paced screening tool in their own environment would help overcome issues related to fatigue and cognitive load discussed under capability barriers. Access to social workers was rarely reported, but was an opportunity enabler for social needs assessment in clinics where this existed.

#### 3.2.5 Motivation (Clinicians)

Several motivation barriers were identified by clinicians. Some clinicians perceived themselves as lacking confidence to appropriately address social needs once they were identified, while others lacked belief in their ability to have these conversations, sometimes due to a lack of familiarity with social needs:

It's sometimes hard to talk about topics that you're either not comfortable with, familiar with, or don't know where to go

clinician 3, nurse

Some clinicians reported a lack of motivation to use a specific social needs screening tool due to insufficient evidence of the value it would provide to patients or clinicians.

I'm just trying to figure out whether it's actually beneficial…. I'm not sure whether I will use something like this and whether it will streamline things

clinician 8, neurologist, interview

Clinicians also identified several motivation enablers. Most clinicians saw addressing social needs in some capacity as part of their professional role; however, the extent of this varied between participants.

Healthcare doesn't exist in a bubble for people…. I feel like it's almost negligent of a clinician not to be aware of what else is going on

clinician 1, allied health

Clinicians held enabling beliefs about the potential benefits of a screening tool, expressing beliefs that it would lead to *consistency and structure (clinician 6, nurse)*, more proactive screening, allow identification and action towards otherwise undisclosed unmet needs, and ultimately improve short‐ and long‐term patient outcomes.

Holistic care was a commonly identified part of the nursing role, which was an additional motivation enabler.

#### 3.2.6 Motivation (Consumers)

There were several motivation barriers identified by consumers. Consumers expressed frustration about previous experiences or potential future experiences of disclosing information that is then not acknowledged and were concerned that a social needs screening tool might result in similar experiences. Consumers also expressed pessimism regarding health or social support services being inadequately resourced to take meaningful action to address social needs. Further, strong privacy concerns were expressed by one consumer related to completing a social needs screening tool.

A key theme identified as a motivation enabler was a desire for unmet social needs to be addressed as a result of disclosure.

### 3.3 Feasibility of Screening for Social Needs

The feasibility of implementing a social needs screening tool in MS care was assessed with themes mapped to six feasibility domains [27] (Table 3). Additional quotes are provided in Supplementary Table 5.

**Table 3:** Feasibility^a^ of implementing social needs assessment in multiple sclerosis care.

Feasibility domain	Theme (clinician)	Theme (consumer)
Acceptability	Acceptable, conditional on associated resources/referral pathways and short administration time	Acceptable, conditional on identified needs being acknowledged and addressed.
	Opportunity cost (neurologists)	Opportunity cost (neurologist appointments)
Demand	Likely to adopt periodically (e.g., every 6 months), conditional on associated resources and short administration time	See Acceptability
Implementation	How: completed by the person with MS prior to the appointment, reviewed in the appointment	How/Where: in own time, at home prior to the appointment, reviewed in the appointment
	When: periodically, not every session	
	Who: Most healthcare professionals	Who: Most healthcare professionals
	Where: in a waiting room or at home, with consideration for safety and privacy	What: Desire for identified needs to be acknowledged, discussed and followed up
Practicality	Requires associated resources, i.e., referral pathways/algorithms	See Adaptation
	Requires some training (e.g., associated manual and education that tool exists)	
	Reinforcement, cultural change	
	Time to review and follow‐up	
	Appropriate staffing	
	Appropriate space for individuals to complete	
Adaptation	Accessible formatting or technology: electronic, own device.	Response options that capture complexity while minimising cognitive load
	Brevity: Single page, or pyramid/expansive structure	
Integration	Could be provided to people with MS to complete with other onboarding/pre‐appointment paperwork	N/A

^a^ Feasibility domains according to the feasibility framework of Bowen et al. [27].

#### 3.3.1 Acceptability and Demand

A social needs screening tool was considered acceptable with a high likelihood of adoption (demand) by clinicians. However, this acceptability and demand was generally conditional on the tool having accompanying resources to signpost or operationalise referral pathways, adequate resourcing for administration, and brevity (i.e., *not going to add too much time [clinician 7]*). Further, clinicians reported that they would be most likely to adopt a screening tool at initial assessment and periodically thereafter, rather than during every review.

I probably would see it [as] something more [completed at] initial screening, and then maybe every six or 12 months.

clinician 3, nurse

For consumers, a social needs screening tool was met with some scepticism, but considered generally acceptable, conditional on disclosed needs being acknowledged and followed up by clinician action.

If there [were] real outcomes to it … maybe I would fill it out.

consumer 5

#### 3.3.2 Implementation and Practicality

Many considerations were raised regarding the implementation and practicality of a social needs screening tool: these included discussion of where it should be completed, which clinician is best placed to employ it, and what is needed to practically implement it.

Clinicians reported a preponderance towards consumers completing a screening tool prior to the appointment rather than in‐person with the clinician, to promote time efficiency and honest disclosure. Pre‐appointment completion of a screening tool was also supported by consumers, who reported that the ability to complete a screening tool in their own time and in their own environment was preferable, as long as it was followed up in their appointment.

As long as you fill it in beforehand and then they actually talk about it

consumer 4

One consumer and two clinicians raised concerns about the barriers to disclosing family violence or abuse and that clinicians should *just ask (consumer 7)* where the person with MS prefers to complete the tool.

When asked which healthcare professional was best positioned to perform social needs screening, clinicians said this depended on the resources of the setting (noting that not all MS care settings have access to a multidisciplinary team) and that most health professionals could do it. Neurologists were commonly seen as the most time‐poor and therefore not well‐placed to assume responsibility for the task of screening for social needs. Consumers told us that assessment of unmet social needs is the role of every healthcare professional, although some consumers expressed concerns about the opportunity cost of neurologists screening for social needs.

Why would we want to be doing it with our neurologist? … I want him to tell me upcoming MS treatments. I want better use of his time.

consumer 6

There were several practical resources described by clinicians as required to implement a social needs screening tool. Associated referral pathways or flow charts to accompany a screening tool were discussed by many clinicians, along with a user manual and education to aid the adoption a screening tool. Resources—including *more staff, more time (clinician 8, neurologist, interview)* and physical space—were identified as practical needs to successful implementation of a screening tool in already busy MS clinics:

I know other clinics around the country are set up with very little time, very little space, very little capacity to do that

clinician 2, nurse

#### 3.3.3 Adaptation and Integration

Specific adaptations to two existing social needs screening tools were discussed. Some clinicians felt all items on a screening tool should be actionable, while others also thought non‐actionable items that provided a holistic understanding of the individual should be included. Two clinicians also reported that using a *strengths‐based (consumer 8)* approach to questioning (which the existing tools lacked) was an important future adaptation.

Consumers described an affinity towards formatting responses that reduced cognitive load, including the use of smiley‐face visual scales which *gave me somewhere to key in without having to find a word (consumer 8)* and provided an opportunity to show *exactly how I feel … without having to really say anything (consumer 3)*. There was a resistance from both clinicians and consumers to oversimplify responses, with a need to capture complexity that was overlooked by binary response options. Clinicians and consumers both identified the importance of using accessible language, formatting and technology in the delivery of a social needs screening tool.

[I would prefer to answer these questions] electronically, with some mode of technology which actually supports [a person with MS]

consumer 1

Integration was discussed, with clinicians suggesting a screening tool could be incorporated into existing pre‐appointment questionnaires that are already sent out, or other onboarding forms at entry into the service.

## 4 Discussion

There is currently no consistent or comprehensive approach to assess or address unmet social needs in MS care in Australia. This poor integration of social and health care hinders optimal health outcomes for people with MS and likely widens inequalities in healthcare and health outcomes [4]. Implementation of a social needs screening tool in MS care was considered conditionally feasible and acceptable.

Lack of knowledge of available resources and referral pathways was reported as a key capability barrier for clinicians to assess unmet social needs, which is consistent with previous literature [35]. Training and education are commonly proposed as potential interventions to address barriers related to these psychological capabilities [26]. Clinicians discussed many ways to address unmet social needs, with the prerequisite of a screening tool with associated resources (e.g., algorithms, flow charts, referral pathways and user manuals) emerging as a strong theme related to practical implementation. Resource toolkits, for example, the WeCan supportive care platform that provides people living with cancer, carers and healthcare providers links to relevant social services [36], have been successfully implemented in Australia. This platform links in well with the Nursing Equity Assessment Tool (NEAT), a brief screening questionnaire that assists cancer nurses to identify unmet social needs in their patients [37]. Young stroke survivors in Australia have also proposed the use of technology to share contemporary resources specific to their needs, including employment, financial and social participation [38]. A similar centralised portal of resources to empower people with MS, carers and clinicians to find adequate support for unmet social needs could assist in filling this identified knowledge gap. Consumers emphasised that clinician action in response to disclosed social needs was crucial; this is consistent with recommendations that social needs screening should not occur without capacity to ensure appropriate referrals to social and/or community services [39], which further underscores the necessity of such resources. Clinicians and consumers reported that good rapport and communication skills were key enablers to having sensitive conversations about unmet social needs, which aligns with previous research [35, 37, 40, 41, 42].

Lack of resources, including time, space and staff, and lack of coordination of care between hospital and community services was a key opportunity barrier to assessing unmet social needs in MS care. Lack of resourcing has commonly been reported as a barrier to implementing social needs assessments in the healthcare setting [35, 37, 41, 42, 43, 44]. Environmental restructuring (e.g., models of care that incorporate a social needs support person) and enablement (e.g., electronic platforms for screening and prescribing) are proposed as potential interventions to address such physical opportunity barriers [26]. Our participants proposed a dedicated, centrally located social worker or occupational therapist (e.g., associated with MS charitable organisations) to provide coordinated support. This is important given that coordination of care between healthcare professionals was highlighted as a major opportunity enabler by many of the participants with MS in our current study but was often lacking in their experience. While it is acknowledged that such interventions would require additional resourcing, these services have the potential to prevent social needs and resulting poor health in the future [45]. Similar models employing supporting clinicians and non‐clinical ‘link worker’ have been successfully piloted in various US and UK healthcare settings [46, 47, 48]; however, to our knowledge, this model has not been piloted in MS care in Australia. This model might resolve the resource and knowledge barriers by streamlining the follow‐up process for identified unmet needs. Extending this model to include telehealth and electronic prescribing might also promote more equitable access to healthcare [49]. Further, virtual assistants present a novel potential approach to providing straightforward functional social support for some individuals, although the implementation and appropriateness to the MS care setting would require careful consideration [50].

A key motivation barrier for clinicians to implement a screening tool was the lack of evidence of value or benefit to patients or clinicians. Previous studies have also suggested that demonstrating evidence of tool sensitivity and improved patient health outcomes is needed to improve clinician buy‐in for social needs screening implementation [35, 37]. A feasibility study of the NEAT social needs screening tool in cancer care showed that nurses reported benefits including faster identification of unmet patient needs, a better understanding of barriers to care and factors that may result in complex care needs, and improved communication with other health professionals and patients [37]. However, evidence to support the benefit of addressing unmet social needs are largely based on data from the United Kingdom [51, 52] and the United States [18, 53]. Given the variation in social and healthcare systems internationally [54], social needs screening and interventions should be trialled within local contexts, and in MS care. It is clear from our data that demonstrating the value and potential outcomes of a social needs screening tool is important for clinician adoption. Therefore, evidence is needed to assess whether better integration of social prescribing in clinical MS care (e.g., by implementing a brief screening tool) improves quality of care, patient health outcomes, healthcare utilisation or economic outcomes over time. If this can be established, interventions such as education (e.g., training packages or resources) or modelling (e.g., through clinical champions [55]) could assist in overcoming motivation barriers of lack of evidence of value and lack of belief in capabilities [26]; this would also capitalise on clinician motivation enablers, including intentions to provide holistic care and to improve patient outcomes.

Participants in our study indicated initial feasibility and acceptability of implementing a social needs screening tool, such as the FUST [30] and Social Determinants of Health Screening Tool [31], in MS care. Small pilot trials of social needs screening tools in Australian clinical populations (including sleep disorders, anxiety disorders, cancer, chronic lung disease, heart failure and diabetes) have previously demonstrated that social needs screening tools are generally acceptable to clinicians and consumers, dependent on the tool being brief, simple and self‐complete and having a clearly stated purpose [30, 31, 37, 56]. Some trials have also highlighted lack of time as a barrier to social needs screening for doctors and support the role of social workers or other healthcare professionals in supporting the integration of health and social care [37, 56].

### 4.1 Future Directions

A recent scoping review demonstrated the variability of social prescribing programs internationally and a lack of social prescribing programs in Australia, which was represented by 3 of the 148 identified studies [57]. Social prescribing models employ various funding models (including government, charity, community‐based organisations and private companies), staffing models (including existing healthcare workers, new link workers, volunteer and student link workers), and settings (including healthcare, community and online) [57]. Further, target populations and non‐medical needs in the described social prescribing models ranged from specific to general. Due to the variability of social prescribing programs, six key planning aspects have been described: (1) identifying the target population, (2) determining the non‐medical needs to be assessed, (3) identifying the support services, (4) determining the setting, (5) determining staffing and training requirements, and (6) determining funding [57].

These key aspects of social prescribing should be considered in the Australian MS care context. Our data suggest that social prescribing for Australians with MS should address their broad range of social needs with specific interventions to provide support for unmet needs; identifying supports through service mapping, an online database, resource toolkits or another up‐to‐date resource are potential methods for identifying support services. Regarding the context, the Australian government has identified the goal of embedding enhanced referral pathways to community services into local health systems as part of their most recent National Preventative Health Strategy, which supports the healthcare setting as an appropriate context in which screening and social prescribing could occur [49]. This might pave the way for a pilot trial of a co‐designed screening tool for use in MS care settings, incorporating feedback from our consumer and clinician focus groups, with associated referral pathways and resources. Regarding staffing, our participants supported a model where a healthcare professional, such as an MS nurse, is dedicated to coordinating social and health care. Next steps would include trialling the feasibility and acceptability of this model of care, or a non‐clinical link worker, and training requirements in the Australian setting.

Finally, the importance of co‐design in social needs screening and social prescribing should not be overlooked. The process of co‐designing social prescribing in a regional area of Australia was recently described [58] and underscores the value of stakeholder input in similar initiatives. A co‐designed approach would help to address the capability barrier reported by consumers in our study related to the cognitive demand of a screening tool, as well as capitalising on the opportunity enabler of providing patients with appropriate time and environments to self‐complete such a tool prior to their initial appointment and periodically thereafter.

### 4.2 Strengths and Limitations

To our knowledge, we provide the first detailed exploration of social needs assessment in MS care, and the barriers and enablers for implementing a screening tool. The study design enabled a comprehensive understanding of the experiences and perspectives of two key stakeholder groups: clinicians working with people with MS, and people diagnosed with MS or caring for people with MS. We used a range of data collection methods which also allowed us to triangulate our findings. The breadth and depth of experience in the research team was another strength of the study, further substantiating the design and analysis with clinical and methodological expertise. To ensure transparency and reporting standards, we adhered to guidelines for reporting qualitative analyses [28].

A limitation of the study was the large representation by female clinicians (73%), and lack of representation from four Australian states/territories (Western Australia, South Australia, Northern Territory and Australian Capital Territory) or rural and remote areas. While this might limit the transferability of the findings, this is partially representative of the current workforce: in 2022, the Australian healthcare workforce comprised 74% females and in 2017, a survey of Australian MS Specialist Nurses showed that South Australia, Northern Territory and Australian Capital Territory made up 7%, 0% and 0% of MS Specialist Nurse respondents, respectively, while Western Australia made up 26% of respondents [59, 60]. Occupational therapists were also identified by participants as being well‐placed to screen for and follow up social needs; however, this clinician group was not represented in our sample. While we were able to capture data through semi‐structured interviews and paired interviews from neurologists and another subgroup of allied health professionals (who were not available for our initial focus groups), we acknowledge that this method may not have captured the same depth of discussion and idea generation as our focus groups and would ideally be treated as a distinct type of data collection. Methodological rigour could have further been improved through more formal intercoder reliability assessment, for example, using percent agreement. Finally, the requirement of participants to attend focus groups via Zoom and speak English potentially precluded input from consumers with more advanced disease, who are non‐English speakers, or who are unable to use technology; factors which might contribute to unmet social needs.

## 5 Conclusion

There is a clear need for better integrated social and MS healthcare. The implementation of a screening instrument offers a systematic approach to identifying unmet social needs and facilitating targeted interventions, and was considered acceptable by our participants, conditional on appropriate resourcing and/or design to ensure identified needs can be addressed. Strategies to addressing key barriers and leveraging the enablers identified in this study, such as co‐designing resources, identifying referral pathways, employing a care coordinator (e.g., a social worker or link worker), and measuring the impact on the health and well‐being of people with MS, will enable future implementation. Addressing unmet social needs through MS care represents a promising avenue for improving comprehensive MS management and reducing inequalities in health outcomes.

## Author Contributions

**Megan R. Hawkins:** writing – original draft, formal analysis, methodology, data curation, project administration. **Yvonne C. Learmonth:** formal analysis, supervision, conceptualisation, methodology, data curation, funding acquisition, writing – original draft. **Isabelle Weld‐Blundell:** writing – review and editing, conceptualisation, methodology, funding acquisition. **Darshini Ayton:** writing – review and editing, methodology, conceptualisation, formal analysis, funding acquisition. **Jodi Haartsen:** writing – review and editing, conceptualisation, methodology, funding acquisition. **Anne Kavanagh:** writing – review and editing, conceptualisation, methodology, funding acquisition. **Tomas Kalincik:** writing – review and editing, conceptualisation, methodology, funding acquisition. **Marlena Klaic:** writing – review and editing, conceptualisation, methodology, funding acquisition. **Claudia H. Marck:** writing – original draft, formal analysis, supervision, conceptualisation, methodology, data curation, funding acquisition, project administration.

## Ethics Statement

Approval was obtained from the ethics committee of The University of Melbourne, Victoria, Australia (2025‐31423‐63802‐3). The procedures used in this study adhere to the tenets of the Declaration of Helsinki.

## Consent

Informed consent was obtained from all individual participants included in the study.

## Conflicts of Interest

The authors declare no conflicts of interest.

## Supporting information

Supporting File 1

Supporting File 2

## Data Availability

Data are not available due to privacy/ethical restrictions.
