# Supplementary material for: Assessing Unmet Social Needs in Multiple Sclerosis Care in Australia: A Qualitative Assessment of Feasibility, Barriers and Enablers
Source: Health Expect. 2026 May 18;29(3):e70691. doi: 10.1111/hex.70691 (PMC13181325; doi:10.1111/hex.70691)
Supplement: Supplementary file 1 — Supporting File 1 [file HEX-29-e70691-s001.pdf]

Focus group discussion guide for MS clinicians

Obtain verbal consent from all participants, start recording.
Introductions, confidentiality (please do not disclose any private information about yourself or others).
Overview of screening tools and summary of pre-reading materials.

Domain	Discussion prompts (main prompts are in bold)
Brief presentation	• [Brief overview of the 2-3 screening tools for social needs most relevant to people with MS in Australia]<br>• **"Now that we have shared some information about social needs and screening for social needs— was there anything that we could explain better or that stood out to anyone?"**
Opening questions	• **"What is your experience with screening for social needs? Do you already screen for these? How are you screening (i.e. informally, using screening tools)? When are you screening (i.e. initial consult, follow-up consults)?"**<br>• **"What do you think of [name of screening tools]? Why?"**
Relevance	• **"How suitable do you think these screening tools are for people with MS? Why?"**<br>• **"What questions do you think are most relevant to social needs of people with MS? In the Australian clinical setting? Why?"**
Comprehensiveness	• **"Tell me about anything important or unique to people with MS that is missing from these screening tools?"**<br>• *Additional prompt if needed: "*Do you think that these screening tools adequately cover the social needs of people with MS?"
Clinical feasibility	• **"How likely are you to use these tools in your daily practice? Why?"** [*acceptability, demand*]<br>• **"How would you incorporate a tool like this into your clinical practice?"** *Additional prompts if needed*: "Whose role do you think it is to administer these screening tools in practice? What role do MS nurses play in this?" [*implementation*]<br>• **When do you ask these? Are these different at different points in patient journey?"**<br>• *Additional prompt if needed*: "How could these tools be incorporated into the systems already in place in your clinic or hospital? What changes would be needed?" [*integration*]<br>• **"What would make it easier for you to use these tools in your practice?"** [*practicality*]<br>• **"If you identified social needs in a patient, what would you do with this information / what next steps would you take?"** (e.g. referral to social worker) [*implementation*]<br>• **"What do you need—whether it is tools, training, or resources—to act on the social needs you identify in your patients?"** [*implementation*]<br>• **Do you feel it is part of your role to understand a patients' social needs?**

Based on Smith et al., content validity questions guided by COSMIN criteria, and feasibility questions guided by Bowen et al.

Focus group discussion guide for MS consumers

Obtain verbal consent from all participants, start recording.
Introductions, confidentiality (please do not disclose any private information about yourself or others).
Overview of screening tools and summary of pre-reading materials.

Domain	Prompt (main prompts are in bold)
Brief presentation	• [Brief overview of the 2-3 screening tools for social needs most relevant to people with MS in Australia.<br>• **"Now that we have shared some information about social needs and screening for social needs— was there anything that we could explain better or that stood out to anyone?"**
Opening questions	• [Reiterate to participants that this focus group is not about their sharing personal story of social needs, but more so about their experiences of completing screening tools]<br>• **"Have you ever been asked about your social needs by your healthcare team? What was that experience like?"**<br>• **"What do you think of [name of screening tools]? Why?"**
Relevance	• **"What questions do you think are most important to ask people with MS? Why?"**<br>• **"Do you think these tools would work well for people with MS? Why?"**
Comprehensibility	• **"Is there anything confusing about these screening tools or anything that doesn't make sense?"**<br>• *Additional prompt if needed:* "How could we make the tools easier to understand?" (e.g. wording of questions, response options, layout)
Comprehensiveness	• **"Tell me about anything important or unique to people with MS that is missing from these screening tools?"**<br>• *Additional prompt if needed:* "Do you think these tools ask about everything that matters when it comes to the social needs of people with MS?"
Clinical feasibility	• **"How would you feel if you were asked to complete one of these screening tools? Why?** [*acceptability, demand*]<br>• **What would make it easier or more comfortable for you to answer these screening tools?"** [*practicality*]<br>• **"How would you prefer to complete these screening tools—at home before your appointment, in the waiting room, or during your appointment? Would you prefer to answer them in writing or in conversation with a health professional?"** [*implementation*]<br>• **"What should / would you want to happen if you complete this screening tool?"** [*implementation/outcome*]

(adapted from Smith et al., content validity questions guided by COSMIN criteria, and feasibility questions guided by Bowen et al.)
