# Supplementary material for: Assessing Unmet Social Needs in Multiple Sclerosis Care in Australia: A Qualitative Assessment of Feasibility, Barriers and Enablers
Source: Health Expect. 2026 May 18;29(3):e70691. doi: 10.1111/hex.70691 (PMC13181325; doi:10.1111/hex.70691)
Supplement: Supplementary file 2 — Supporting File 2 [file HEX-29-e70691-s002.docx]

Supplementary Table 1. Clinician and consumer experiences and views on current social needs assessment in MS care (with key quotes)

| Theme (clinician) | Key quotes | Theme (consumer) | Key quotes |
| --- | --- | --- | --- |
| Informal and inconsistent screening and follow up | *“I guess we here at [health service] don’t have a specific tool for this specific reason... however we do address a lot of those issues in our interviewing process” (clinician 6, nurse)*  *“You might have one or two nurses who were very good at doing this stuff and the rest of them who... unless they can see that they need to be doing this, they’re not going to do it” (clinician 10, allied health)* | Informal and inconsistent screening and follow up/care-coordination | *“[I haven’t been asked about social needs] in depth... my neurologist sort of knows who I live with and knows that I'm working... she's never really asked, like, do you have enough money to buy food like, do people in your household actually help you?” (consumer 4)*  *“I knew about NDIS and all the things that I could get was because of previous experience. I hadn't heard anything from the neurologist.” (consumer 3)* *“If you end up in hospital with a flare... you've got the nurses... the social worker gets involved, that's when those questions sort of come out... They involve the OT [occupational therapist], they involve the support coordinator [someone who helps NDIS participants identify and access services], they involve all these people ‘cause you’ve got a multidisciplinary team in the hospital, [as] opposed to a singular person, which can actually tackle these types of things. And they've got more time because you are sitting there... you've actually got that physical time with, not just the neurologist, but the neurology team and also the nursing staff, and they can engage with other areas in the hospital to get your needs seen better.” (consumer 5)* *"I find everyone passes the buck to the next person. So my neurologist always says to me, ‘Your GP needs to take care of these issues’. When I speak to my GP... he says to me, you're going to have to discuss medications and treatments with your neurologist cause that's his area of expertise” (consumer 6).* |
| Reactive and insufficient | *“Certainly it is our, any healthcare professional role... however are we doing it sufficiently? Probably not” (clinician 4, nurse)*  *“Wouldn't it be amazing if, big picture dreaming, if [neurologists] could see that they won't have to deal with someone crying in their office [saying] I'm homeless, if we can be proactive and get on to this early, so this is why the [screening] tool would be really good.” (clinician 10, allied health)* | Self-advocacy (speaking up for myself and my needs, access to information to make informed decisions) | *“I was able to advocate for myself because of my background... what I saw in the waiting room was a number of people that I'm looking at them thinking... there is no way you could advocate for this for yourself” (consumer 7)*  *“I have to keep pushing everybody to do stuff and then I think if I was more advanced than what I am now... what would happen? I'd be a bit scared because who would look after me or look out for my best interest if I couldn't do it for myself.” (consumer 6)* |
|  |  | Equity issues related to rurality and eligibility for services | *“From [city] I had to travel to [city in a different state 950km away] for that... I would say that it would be very rare that anyone living in a regional location would have access to an MS clinic.” (consumer 1)*  *“It seems to be a zoning type thing or a location-based thing as well. I'm lucky enough to be at the [large tertiary hospital] and I get all that I get.” (consumer 5)* |
|  |  | Perceived lack of clinician interest | *“I get a neurologist who looks at an MRI and that’s it. He just writes a referral every 12 months, there’s no discussions, no talking or anything” (consumer 1)*  *“The holistic stuff, a lot of doctors don’t have time for it. I understand that they’ve got their study and things that they’re allowed or authorised to approve, but holistic is the only thing that helped me a lot” (consumer 6)* |

Supplementary Table 2. Barriers and enablers to social needs assessment in MS care, mapped to the COM-B^a^ capability domain (with key quotes)

| Domain: Capability | Themes (clinician) | Key quotes | Themes (consumer) | Key quotes |
| --- | --- | --- | --- | --- |
| Barriers | Lack of knowledge of available resources | *“Asking the question is rather easy once you're trained in delivery, but then knowing what to do with that and where to lead people with that, at this point in time, I wouldn't feel confident. And maybe that's even influencing me asking” (clinician 1, allied health)*  *“Hospital is hospital focused – there is little knowledge or referral to community from the outpatient clinic.” (clinician 11, allied health, paired interview)* | Cognitive capacity (fatigue, anxiety) | *“I’m already anxious going to an appointment, because I know that this is my one time in six months that I have to explain everything... perhaps this [screening tool] could be given where people have the time, and fill it out in their own capacity” (consumer 7)* |
| Enablers | Patient clinician rapport (including longevity and continuity of care) | *“That's also the benefit of building these longer-term relationships and the importance of checking in over time, of asking, how's the job, how's family, how's things going? It might seem a bit mundane sometimes, but they're very, very important questions for, if not today, but tomorrow” (clinician 6, nurse)* | Self-advocacy  (speaking up for myself and my needs, access to information to make informed decisions) | *“I'm aware through a professional basis of what's offered on NDIS, but it certainly was never educated to, it was never provided” (consumer 15)*  *“I'm fortunate enough to understand and know about support workers [from previous professional experience]… but a lot of people don’t know this stuff” (consumer 3)* |

^a^Barriers and enablers mapped to COM-B framework(1)

Supplementary Table 3. Barriers and enablers to social needs assessment in MS care, mapped to the COM-B^a^ opportunity domain (with key quotes)

| **Domain: Opportunity** | **Themes (clinician)** | **Key quotes** | **Themes (consumer)** | **Key quotes** |
| --- | --- | --- | --- | --- |
| **Barriers** | Lack of resources (time, space, staff)    Coordination of care | *“Clinicians don't like to go beyond their speciality because if you start digging, you need to act on it, and you don't have resources.” (clinician 4, nurse)*  *“We don't have in-house social work anymore at [our organisation] ... I don't know off the top of my head like how I would just refer them into a social worker.” (clinician 3, nurse)*  *“In movement disorder clinics, they have social workers, in cognitive dementia and memory clinics, they have social workers. Yet, in MS clinics, it's something that [is] not present in any of them.” (clinician 10, allied health)* *“Because of some of the sensitivity of the questions, for some people, they might not want to complete it in the home environment, like if they need assistance from a family member to assist them with completing them.” (clinician 5, allied health)* *“The community-hospital link is not very good” (clinician 11, allied health, paired interview).* | Lack of resources (access to MS nurses)  Lack of rapport | *“I have looked into [finding an MS nurse] and there's really not many, like, for where I live, there's maybe two at the hospital, but they look after the whole [regional area]... they have, like, over 1000 patients” (consumer 4)*  *“I very much kind of just keep to myself now about it because with MS, who, if I talk to professionals, certain professionals, it really feels like I'm talking—bleh bleh bleh bleh—I'm filling out things, but I'm not really heard. There's nothing, there's nothing heard.” (consumer 3)* |
| **Enablers** | Coordination of care | *“We do have a very close working relationship with [MS organisation], like [the organisation] actually does come to our clinic and we've got a multidisciplinary team meeting after clinic and this is where a lot of this stuff gets raised.” (clinician 8, neurologist, interview)* | Flexible, self-paced completion  Access to multi-disciplinary team | *“[I need to] take it in and to spend more time, and I would be happily do a lot more thorough one, if I was given something via e-mail and I didn't have to print it” (consumer 3)*  *“There are social workers within, well, I know they are within [my MS clinic] that coordinate all the services into one. So maybe for me that's what I would prefer.”(consumer 3)* |

^a^Barriers and enablers mapped to COM-B framework(1)

Supplementary Table 4. Barriers and enablers to social needs assessment in MS care, mapped to the COM-B^a^ motivation domain (with key quotes)

| Domain: Motivation | Themes (clinician) | Key quotes | Themes (consumer) | Key quotes |
| --- | --- | --- | --- | --- |
| Barriers | Lack of belief in capability (to have conversation or provide solutions)  Lack of evidence of value | *“It's almost like someone answers yes to this question, what are the support services and where can you direct them as a clinician? Or who do you reach out to? I feel like that is definitely what I would feel uncomfortable with, because I'm not confident in that space.” (clinician 1, allied health)* *“It's sometimes hard to talk about topics that you're either not comfortable with, familiar with, or don't know where to go. It’s not unlike asking about suicidal ideation. Sometimes it's feeling like, ‘Oh, I can do this, I've got this’. I obviously understand the bare basics of some of these [social needs], but it's not something I'm familiar with having conversations with people about so I suppose [there is] a risk of ‘Am I going to do it wrong?’” (clinician 3, nurse)*  *“I'm just trying to figure out whether it's actually beneficial compared to what we're already doing... I'm not sure whether I will use something like this and whether it will streamline things” (clinician 8, neurologist, interview)* | Scepticism about clinician follow-up  Scepticism about availability of resources (belief about consequences,  pessimism)  Privacy concerns | *“What are they going to do with it is my question. I’ve spent 35 years filling in forms. What happens to those forms?” (consumer 8)*  *“[I] just don’t think there’s the capacity there. I don’t think the medical system’s got the capacity to take any notice of it.” (consumer 5).*  *“[MS not-for-profit organisation are] doing their best to help… but their hands are tied with how much they can help me” (consumer 6)* *“You’re also filling out personal details and stuff like that on paper, and all of a sudden you've got a privacy concern…you don't know where the data is going… if something happens with a breach… the ramifications of that is just so high” (consumer 5).* |
| Enablers | Part of health professional role to refer and screen  Structure and consistency  Improved patient outcomes / Benefits of proactive screening and data collection  Intentions (holistic care, actioning needs) | *“Healthcare doesn't exist in a bubble for people, people don't present with just a condition or just a diagnosis, and I feel like it's almost negligent of a clinician not to be aware of what else is going on” (clinician 1, allied health)* *"Having a structured, systematic approach means that we're picking up these things early and maybe we can refer more patients to [MS organisation], which is always a good thing I think and provide them more holistic type approach.” (clinician 8, neurologist, interview)* *“If we consistently [collect] this kind of data, we can gather huge data to come up with [a] brighter, bigger project and plan for the patient. So not only short term [but] for long term definitely it would help.” (clinician 4, nurse)* *“We nurses… we believe in providing holistic care, so part of holistic care, we do ask all these questions” (clinician 4, nurse)* | Desire for disclosed social needs to be addressed | *“As long as you fill it in beforehand and then they actually talk about it or, like we've all really said, that they do something with that information”-(consumer 4)*  *“And if these screening tools were given to people and we could fill them out, and then we knew... it actually went somewhere, like it made a difference”(consumer 3)* |

^a^Barriers and enablers mapped to COM-B framework(1)

Supplementary Table 5. Feasibility ^a^ of implementing social needs assessment in multiple sclerosis care (with key quotes)

| Feasibility domain^a^ | Themes (clinician) | *Key quotes* | Themes (consumer) | *Key quotes* |
| --- | --- | --- | --- | --- |
| Acceptability | Acceptable, conditional on associated resources/referral pathways and short administration time  Opportunity cost (neurologists) | *“I [would use a screening tool] if it comes with the preamble and some resources attached to help me deliver it and deliver support” (clinician 6, nurse)*   *“I feel like as a neurologist and someone who's going to make those decisions about MS care, which treatment etcetera, those are more important for me to address. So, so yeah, if I need to answer and prioritise questions, I will take more of the clinical judgement as the priority for myself and leave those ideas for someone else who is better and more knowledgeable than me [in social needs management]” (clinician 9, neurologist, interview)* | Acceptable, conditional on identified needs being acknowledged and addressed.  Opportunity cost (neurologist appointments) | *“I'd say to them what by filling out this, what is it going to? [Are] there any advantages to it? If it's just a bit of paper for the sake of a bit of paper? Yeah, I'm not going to do it. But if there [were] real outcomes to it and stuff like that, maybe I would fill it out.” (consumer 5)* *“Why would we want to be doing it with our neurologist? Like, if I see my neurologist, I want his expertise… He's only in there with me for a few minutes, I don't want to be going through any of this stuff [about my social needs] with him, I want him to tell me upcoming MS treatments. I want better use of his time.” (consumer 6)* |
| Demand | Likely to adopt periodically (e.g. every 6-24 months), conditional on associated resources and short administration time | *“I probably would see it [as] something more [completed at] initial screening, and then maybe every six or 12 months.” (clinician 3, nurse)*  *“Ideally [a screening tool would be completed] right at the start… And in terms of how often to do it after, maybe every couple of years? Every year, every couple of years?” (clinician 13, neurologist, interview)* | Demand for more holistic approach | *“I just think that there should be more of an understanding like we were saying before that holistic, with, with medicine, I don't think medicines is the answer for everything.” (consumer 14)* |
| Implementation | How: completed by person with MS prior to appointment, reviewed in appointment.  Who: Most healthcare professionals (see also Acceptability: Opportunity cost)  When: periodically, not every session  Where: in a waiting room or at home, with consideration for safety and privacy | *“I think self-administration is fine, with the prompts that will then need to be checked by somebody by a human, and will then need to be actioned. And if OTs are available, they are ideal. If social workers, they're ideal. If you have none of those, the nurses are ideal. If you have a doctor in [regional area], and that's all you’ve got, they're ideal.” (clinician 11, allied health, paired interview)*  *“We would be using it for initial or 6 to 12 months... we have a lot of people who are coming to us weekly, fortnightly, so you certainly just wouldn't need to use it with them every time” (clinician 7, allied health)*  *“I think you'll get the most honesty if it's self-completed, and privately self-completed, with nobody else around.” (clinician 2, nurse)* | How/Where: in own time, at home prior to appointment, reviewed in appointment.  What: Desire for identified needs to be acknowledged, discussed, and followed up.  Who: Various healthcare professionals (see also Acceptability: Opportunity cost) | *“As long as we get to do it in our time… cause cognitively sometimes I'm just way too tired and it’s just I have to read it 1000 times.” (consumer 3)*  *“As long as like you fill it in beforehand and then they actually talk about it or they do something with that information, rather than just keep it in a file somewhere at the back of their office.” (consumer 4)* *“I think most appropriately, probably a nurse [should ask social needs questions]” (consumer 4)* *“Interviewer: Which healthcare professionals do you think should ask you about social needs? Should that be your GP, MS Nurse, a neurologists, allied health? Who should it be?*  *Consumer 8: All of the above… I think you’ve got to start with things like GP. Social workers are supposed to do some of this”(consumer 8)* |
| Practicality | Requires associated resources i.e. referral pathways/algorithms  Requires some training (e.g. associated manual, education that tool exists)  Reinforcement, cultural change  Adequate resourcing required (time to review and follow-up, staffing, space to complete) | *“The way I would see some of this working is through a flow chart that almost links to every question and says if that was answered this way then it should trigger that action.” (clinician 11, allied health, paired interview)*  *“If you don't tell people it exists and how to use it and how to make the most of it, you're going to lose some of the power of it.” (clinician 6, nurse)* *“It's about changing the culture, especially in MS arena… [We need] reinforcement, constant reinforcement. And we need to see value in it” (clinician 4, nurse)* *“Interviewer: What would help you implement these tools?*  *Clinician 8: Maybe a more private waiting room… More time. More staff.” (clinician 8, neurologist, interview)* *“I know other clinics around the country are set up with very little time, very little space, very little capacity to do that, reduced nursing support and resources. We're fortunate here, I think we'd be able to manage it, but I think it would be a real challenge at some places.” (clinician 2, nurse)* | Requires time to discuss during appointment, if necessary | *“Maybe the appointment [with a GP or MS nurse] is more centred around that” (consumer 4)*  *“As long as there was like time to talk about as well. Not just fill it out while you're waiting in the foyer, and then don't even mention it in the appointment.” (consumer 4)* |
| Adaptation | Strengths-based  Accessibility (formatting, language, technology)    Brevity: Single page, or pyramid/expansive structure | *“It definitely is not hopeful, it's deficit based, and I would also love to see somewhere in the tools adapted to MS, ‘Where are you heading, and what's stopping you from getting there?’ Because the whole point of MS care is to get them to a space where they can live a life that's worth having, for them, so why not build that into the screening tool?” (clinician 11, allied health, paired interview)*  *“Accessibility across all fronts [is important]. So language and literacy levels… Is there a recording version that you could play for someone that they could answer to? Or is there a soft copy e-version that people can click or can it be done by text message or something like that?”(clinician 3, nurse)* *“For me [my dream tool] would be like a kind of a pyramid version in the sense of like, you know, could you give me like 3 questions to ask and if something there flag something, then I go deeper and then I go deeper and then I go deeper, I think that would be the best.” (clinician 3, nurse)* | Response options that capture complexity while minimising cognitive load  Accessibility (formatting, technology) | *“You can't just have yes or no. You need yes, no, maybe. Or yes, no, sometimes. Or yes, no, and then after ‘sometimes’ leave the line for people to comment. I don't think anything's black and white.” (consumer 7)*  *“[I would prefer to answer these questions] electronically, with some mode of technology which actually supports [a person with MS] maybe an iPad” (consumer 1)* |
| Integration | Could be provided to people with MS to complete with existing onboarding/pre-appointment paperwork | *“When we have people come into our clinic, we already send them different questionnaires to complete. So if we included this one as another one to complete, it would kind of capture a bit more data for us” (clinician 7, allied health)* | Triggered during diagnostic process | *“Could we integrate [social needs screening] into the diagnosis process so once a person is diagnosed… the neurologist then triggers someone to contact us… ideally an MS nurse, if we don't have an MS nurse, a social worker, and they literally sit and go, here's the groups that you can connect to. (consumer 1)* |

^a^ Feasibility domains according to the feasibility framework of Bowen et al(2)

References

1. Michie S, van Stralen MM, West R. The behaviour change wheel: A new method for characterising and designing behaviour change interventions. Implementation Science. 2011;6(1):42.

2. Bowen DJ, Kreuter M, Spring B, Cofta-Woerpel L, Linnan L, Weiner D, et al. How we design feasibility studies. Am J Prev Med. 2009;36(5):452–7.
